# A Precision Medicine Tool for Patients With Multiple Sclerosis (the Open MS BioScreen): Human-Centered Design and Development

**DOI:** 10.2196/15605

**Published:** 2020-07-06

**Authors:** Erica Schleimer, Jennifer Pearce, Andrew Barnecut, William Rowles, Antoine Lizee, Arno Klein, Valerie J Block, Adam Santaniello, Adam Renschen, Refujia Gomez, Anisha Keshavan, Jeffrey M Gelfand, Roland G Henry, Stephen L Hauser, Riley Bove

**Affiliations:** 1 Department of Neurology UCSF Weill Institute for Neurosciences San Francisco, CA United States; 2 Plain Language Health San Francisco, CA United States; 3 Child Mind Institute New York, NY United States

**Keywords:** human-centered design, mobile phone, personal health record, participatory medicine, visualization in eHealth, human factors

## Abstract

**Background:**

Patients with multiple sclerosis (MS) face several challenges in accessing clinical tools to help them monitor, understand, and make meaningful decisions about their disease course. The University of California San Francisco MS BioScreen is a web-based precision medicine tool initially designed to be clinician facing. We aimed to design a second, openly available tool, Open MS BioScreen, that would be accessible, understandable, and actionable by people with MS.

**Objective:**

This study aimed to describe the human-centered design and development approach (inspiration, ideation, and implementation) for creating the Open MS BioScreen platform.

**Methods:**

We planned an iterative and cyclical development process that included stakeholder engagement and iterative feedback from users. Stakeholders included patients with MS along with their caregivers and family members, MS experts, generalist clinicians, industry representatives, and advocacy experts. Users consisted of anyone who wants to track MS measurements over time and access openly available tools for people with MS. Phase I (inspiration) consisted of empathizing with users and defining the problem. We sought to understand the main challenges faced by patients and clinicians and what they would want to see in a web-based app. In phase II (ideation), our multidisciplinary team discussed approaches to capture, display, and make sense of user data. Then, we prototyped a series of mock-ups to solicit feedback from clinicians and people with MS. In phase III (implementation), we incorporated all concepts to test and iterate a minimally viable product. We then gathered feedback through an agile development process. The design and development were cyclical—many times throughout the process, we went back to the drawing board.

**Results:**

This human-centered approach generated an openly available, web-based app through which patients with MS, their clinicians, and their caregivers can access the site and create an account. Users can enter information about their MS (basic level as well as more advanced concepts), visualize their data longitudinally, access a series of algorithms designed to empower them to make decisions about their treatments, and enter data from wearable devices to encourage realistic goal setting about their ambulatory activity. Agile development will allow us to continue to incorporate precision medicine tools, as these are validated in the clinical research arena.

**Conclusions:**

After engaging intended users into the iterative human-centered design of the Open MS BioScreen, we will now monitor the adaptation and dissemination of the tool as we expand its functionality and reach. The insights generated from this approach can be applied to the development of a number of self-tracking, self-management, and user engagement tools for patients with chronic conditions.

## Introduction

### Background

Delivering actionable clinical tools into the hands of patients and clinicians represents a major unmet need for the delivery of precision medicine for complex diseases such as multiple sclerosis (MS). MS is a chronic, inflammatory, and neurodegenerative disease characterized by onset typically during young adulthood, a protracted and heterogeneous course, variable impairments across multiple functional domains, and variable response to medications [1]. Digital tools help to track this heterogeneity and inform decision making [2] These include digital tools that create research cohorts and registries (eg, iConquerMS [3] and The North American Research Committee on Multiple Sclerosis [4]) and help patients connect with one another and collect and share information (eg, PatientsLikeMe [5], SmartPatients [6], and Facebook groups [7]). In health care settings without a single-payer system or a national electronic health record (EHR), there is a further need for a data collection system to allow patients to collect essential information about their MS course as they navigate multiple providers and EHRs over time. Within this landscape, we aimed to create a freely available platform through which patients can enter basic MS measurements, visualize their course, and access actionable research tools curated by clinicians to inform their decision making.

### Open MS BioScreen

In 2013, the University of California, San Francisco (UCSF) Multiple Sclerosis Group announced the *UCSF Multiple Sclerosis BioScreen* [8]. The MS BioScreen is *a data infrastructure platform* that gathers all relevant MS data from different sources, including clinical, imaging, and biomarker information; visually represents the disease course of an individual with MS from a front-end interface; and frames this course within the context of a large cohort of patients treated according to contemporary standards. The goal is to inform more precise clinical decisions and by providing clear information and decision aids, to empower patients to participate more actively in their clinical care. This work received key support from the Patient-Centered Outcomes Research Institute and the Conrad N. Hilton Foundation. However, this first prototype was developed as a clinician-facing, tablet-based tool and hence was not accessible beyond the limited group of patients followed at UCSF. Due to the limitations of tablet storage and connectivity, the MS BioScreen was not used after the pilot phase.

To expand the reach of our precision medicine solution, we aimed to develop the Open MS BioScreen [9]: a publicly available web-based app, free of commercial interest, that allows users within or beyond highly specialized academic care settings the opportunity to enter data on their condition; obtain a richly contextualized, digestible, and actionable predictive output; and participate in a shared decision-making process. We included *open* in the title as a key concept because the platform is freely available for anyone in any location to create a profile and enter their MS measurements, without requiring a fee or any user validation process.

Here, we describe the human-centered design approach that we applied throughout the development process, an approach that focuses on the usability and needs of those the tool is meant to serve in addition to the specific theoretical framework that was applied as an underpinning to the platform content.

## Methods

### Human-Centered Design Approach

App development of the Open MS BioScreen began in March 2017 and is ongoing. We applied the concept of human-centered design [10,11], an approach that focuses on the usability and needs of those the tool is meant to serve. We included a variety of key stakeholders: individual patients, patient support groups, clinicians, technology consultants, and institutional groups, and we integrated their feedback continuously throughout the development of the app. In the context of chronic disease, the application of human-centered design represents an opportunity to address key underlying provider and patient gaps that obstruct improved health outcomes. As previously described [11], to bridge the knowing and doing gap between evidence-based provider recommendations and patient implementation, solutions must wield individualized and actionable goals that can be addressed within real-world limitations. Without involving key stakeholders in the development, promising technology-leveraged management platforms have difficulty achieving clinical improvement (eg, type 2 diabetes [12] and cardiac rehabilitation [13]). In contrast, tools incorporating human-centered design demonstrate positive effects for specified outcomes across diverse populations [14], including caregivers of patients with Alzheimer disease [15], patients managing chronic obstructive pulmonary disease [16], and in pain management [17].

**Figure 1:**
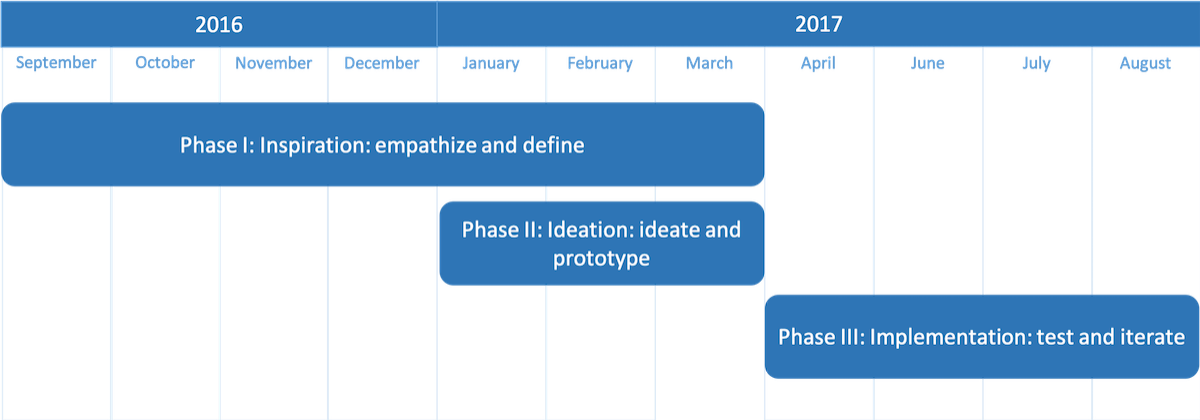
The human-centered design process for Open MS BioScreen.

As outlined in Figure 1, we applied the three phases of human-centered design [11]: (1) inspiration (empathize and define), (2) ideation (ideate and prototype), and (3) implementation (test and iterate).

### Team and Stakeholders

The *Open MS Bioscreen* team *at* UCSF includes a clinical lead (RB), developer (ES), back-end data consultants (AS and AR), technical consultant (AL), radiology consultant (RH), compliance and logistical assistant (WR), designer (AB), students, and research assistants. This group leveraged the expertise of the broader group of UCSF-based MS researchers (the UCSF Expression, Proteomics, Imaging, Clinical [EPIC] longitudinal study [18,19]) as well as team experience with other tools developed through the UCSF MS BioScreen project, namely, Sutter Health MS Share [8] and UCSF BRIDGE [20].

#### Health Literacy Expert

A health literacy consultant with expertise in chronic conditions, including MS, was engaged to provide individual feedback on solution design and patient interview protocols as well as guide content creation using input gleaned from patient focus groups.

#### Key Stakeholders

##### Patients With Multiple Sclerosis

Through convenience sampling, we identified 50 adults with MS who participated either in individual interviews with patients identified by participating clinicians or approached by the study team in the clinic waiting room and at an MS fundraising walk (n=15, often accompanied with a partner, friend, or caregiver) and focus groups (3 groups comprising 6, 8, and 21 people; 2 community-based [National Multiple Sclerosis Society, NMSS, support groups] and 1 UCSF-based). An additional 24 participated in the validation of patient-reported MS duration and treatment and provided individual feedback.

##### Clinicians

Through convenience sampling, we identified 6 UCSF-based MS consultants, as well as 2 external neurologists (1 MS and 1 general), who were included through phases I and II.

##### Advocacy Group

Representatives from the NMSS, a primary organization for MS advocacy and research in the United States, were consulted in phase 1 (N=1) and phase 3 (N=3) to advise on the overall goals of the Open MS BioScreen, clarity and relevance of the content to MS patients, and any ethical or other considerations anticipated.

##### Industry Representatives

This convenience sample of representatives who had approached the MS BioScreen team during the development phases included representatives from 2 biotech companies seeking to develop patient-facing technical solutions for the delivery of MS precision medicine (1 pharmaceutical and 1 biotechnological) as well as 3 experts in the delivery of advanced magnetic resonance imaging (MRI) solutions to patients. The role of input from these representatives was to better delineate the value proposition of an openly accessible, freely available platform within the context of current digital health efforts; however, the platform was not designed to collect data of commercial relevance.

### Theoretical Underpinning

**Figure 2:**
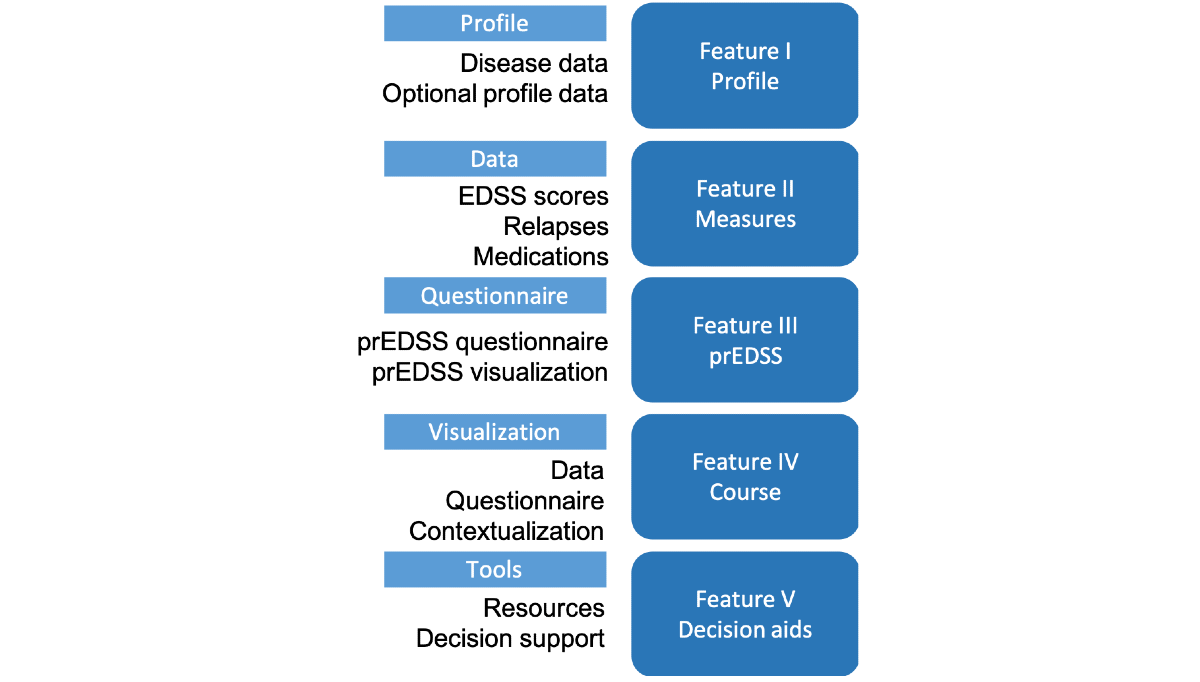
Open MS BioScreen features. EDSS: expanded disability status scale; prEDSS: patient-reported expanded disability status scale.

Although the design process was iterative and human-centered, we identified a priori several key underpinnings of the platform, namely, Open MS BioScreen should comprise 5 features: (I) profile, (II) measures, (III) patient-reported expanded disability status scale (prEDSS), (IV) course, and (V) decision aids, as outlined in Figure 2. Finally, the user can represent a patient, a patient’s proxy (friend, spouse, partner, child, and caregiver), or a clinician [21].

Feature I allows a user to create a profile about themselves. The profile contains required information: month and year of birth and age of onset of MS. In addition, users can add optional information: gender, MS type, city, country, education, employment, race, smoking history, and spinal tap results.

Feature II is used for patient-reported basic MS measures. Users can enter in their standard disability scores (expanded disability status scale, EDSS) [22] results and date of results. Relapse dates can be reported and stored. Additionally, users can enter their MS medication types and dates.

Feature III focuses on 1 specific measure, the prEDSS questionnaire. The prEDSS feature allows a user to answer a series of questions to generate a prEDSS score and visualization and can be used when patients do not have access to a clinician’s objective EDSS score.

Feature IV is a data visualization component that brings in data from features I-III. The patient course is plotted in a single interactive view, allowing the user to see all their MS measurements plotted along with their prEDSS scores. Users can optionally display their course in context with our contextualization cohort to see how their MS course compares.

Feature V is a set of many evolving features. Here, we take relevant and new MS research findings and translate them into interactive and actionable features that serve as decision aids regarding if and when to start MS therapies and how to balance the risks and benefits of treatment approaches. We also link it to other tools that could be useful.

### Phase I—Inspiration: Empathize and Define

The goal of this phase was to define the problem we were trying to solve. To accomplish this, between September 2016 and March 2017, one-on-one in-depth in-person interviews were conducted with clinicians, MS patients (and their friends, families, and caregivers), and industry and advocacy experts. Interview transcripts and questionnaire responses were then reviewed and parsed into key *insights* (Tables 1 and 2).

We gathered information from clinicians, patients, partners and through market research.

1. Clinician interviews: We interviewed 1 MS expert (2 hours) and shadowed 5 others in the clinic (one 4-5 hour session each) to observe the interaction and document the information provided. As part of the observation, we talked with the patients to develop an understanding of them, their MS, and how they stay informed. Following the clinical observations, all interviews focused on the tools available to clinicians to monitor their patients (eg, what types of patient-centered data were missing from the clinical EHR?), decision support tools available to them in routine clinical encounters (eg, contextualization or predictive algorithms readily available and would they be useful), and unmet needs (eg, what types of visualizations would help them communicate risk with patients?).
2. Patient interviews: In total, 12 individual patients were interviewed using semistructured questionnaires, developed with input from the health literacy expert. Interviews lasted 30-60 min and asked patients general questions about their MS and their use of tools to understand and track their condition outside of clinical encounters (Multimedia Appendices 1 and 2).
3. Interviews with technology and advocacy partners: These general interviews lasted 30-120 min and were related to the availability of other tools on the market, relative strengths, and unmet needs.
4. Comparative analysis of existing technological solutions: Here, our team researched other technical solutions available to patients with MS and their clinicians to track their own course and access relevant algorithms and visualizations that empowered them to participate in their own care. To accomplish this, we used the Google search engine, the iOS App Store, and the Google Play Store and used the search terms “multiple sclerosis”
  or
  “MS” and the terms “symptom tracker,” “monitor,” “tracker,” and “algorithm.” From these results, rather than reporting an exhaustive list, we selected tools intended to highlight desired features, possible redundancies/overlap, as well as technological or other pitfalls of existing tools.

**Table 1:** Patient interview insights.

Insight	Patient input
Clarity	Does not want to be inundated with information Wants to reclaim control of their disease—too little clarity, not enough power in their hands Currently feels like there are no clear answers given, feels alone in trying to understand Wants information that is easy to understand and translate between centers Can be medically illiterate at times—feels they have a lot of misconceptions
Data collection and visualization	Tracks in everything in a notebook—filled with other information Wants to see scores
Personalized	Tracking their own progress and treatment efficacy, balanced with their own preferences and needs
Trajectory	Wants to know if they are getting better or worse Wants to know 5-year outlook Constantly playing mind games to stay positive
Comparison	Would like to see other patient data without communicating with them Wants to meet other people with the same progression Wants to gain more perspective about other people with MS^a^
Treatment	Wants to know that the treatments they are getting are useful or the best for their disease
Resources	Wants more information about specific areas of interest—genetics, therapies, and education about MS Wants lots of educated opinions
Time with specialist	Prefers time spent with doctor to time spent researching on their own Does not know what to contact their neurologist about Will not go to the doctor until it is unbearable Wants a printout from every visit

^a^MS: multiple sclerosis.

**Table 2:** Comparative analysis of existing technological solutions: illustrative examples.

Tool evaluated	Strengths	Limitations	How strengths and limitations shaped our phase II process
UCSF^a^ MS^b^ BioScreen [8]	Integrates clinical, MRI, and biomarker data in 1 coherent view	iOS app Not real time; depends on data extracted from study registry or EMR^c^	Enabled patient-entered data Web-based platform preferable
Data living in Advancing Patient-Centered Excellence (UCSF Epic-based EMR)	Clinician-validated Common EMR system allows for aggregation of patient data across multiple sites and institutions	As of 2019, no existing algorithms to extract all key data available from a patient’s entire MS history (in the United States) Difficult for the layperson to identify salient metrics	Build a patient-facing app
MyMSandMe [27]	Patient engagement resource with an active forum Medication diary	No longer supported on iOS or Android platforms	Web-based platform preferable
myMS [28]	Connect to 23andMe genetics Contextualization of personal data Ability to self-report metrics Ability to view MRIs^d^ in-app Tasks and questionnaires available in-app	iOS app Significant involvement with a private company (23andMe)	Genetics not yet actionable Web-based platform preferable Fewer metrics, more relevant to patient clinical picture Enable patient-determined EDSS^e^
Specific activity or symptom trackers (eg, Fitbit)	Ubiquitous Passive data collection and friendly user interface encourage personalized goal setting and engagement	Limited lifecycle Cost Do not integrate with other clinical data	Agility—allow inclusion of data derived from these trackers without needing to integrate with any 1 device or vendor
PatientsLikeMe [5]	Free to use Widely used	Heavily funded by pharmaceutical industry No specific clinical decision support aides	Remain clinically focused to aid key decision-making points in MS history
Swedish MS registry [29]	Presents clinically meaningful data in a user-friendly interface Clinicians/providers contribute validated data to individual profiles	Not available in the United States	Longitudinal visualizations
Floodlight [30]	Validated metrics of MS-related function Smartphone app	Data sent to pharmaceutical company Not available for all smartphone platforms	In the future, enable integration with wearables Enable manual entry of MS functional composite metrics
Aby [31]	Connect with MS experts Guided PT^f^ programs Apple Health integration	Data sent to pharmaceutical company	Links to various sources of clinical expertise
BeCare Link [32]	Tracks symptoms over time by recording a host of unsupervised tests (25’ walk, TUG^g^ test, vibratory sensitivity, etc) Reports proprietary EDSS and MSFC^h^	Testing can take several hours No clinical or clinician-validated data from the EMR	Enable patient-determined EDSS and relapses

^a^UCSF: University of California, San Francisco.

^b^MS: multiple sclerosis.

^c^EMR: electronic medical record.

^d^MRI: magnetic resonance imaging.

^e^EDSS: expanded disability status scale.

^f^PT: physical therapy.

^g^TUG: Timed Up & Go.

^h^MSFC: multiple sclerosis functional composite.

### Phase II—Ideation: Ideate and Prototype

At the completion of phase I, in January 2017, and as detailed below in the *Results* section, we defined the problem that we wanted to solve as: “People with MS are faced with a lot of variables, numbers, and changes, and it is hard to keep track of everything.” In phase II, our goal was to develop mock-ups. Therefore, we began to come up with solutions to this problem: how best to allow users to enter and visualize all their data and incorporate research findings and additional resources together in a cohesive and actionable way, using tools that are sustainable, low cost, and would not require payment on the part of patients. We met weekly to discuss how best to collect and visualize patient data. Phase II meetings lasted for an hour and focused on determining the user interface for data collection and visualization. Methods for idea generation included brainstorming options, whiteboarding, and searching for ways other people visualized similar problems. Phase II meetings started in January 2017 and lasted until March 2017.

The most promising ideas were moved to the prototype, using tools including Balsamiq [23] for creating wireframes and Invision [24] for creating click-through mock-ups. These wireframes and mock-ups were then shown, in a second round of interviews, to clinicians and patients with MS for further feedback. At this stage, the health literacy expert was re-engaged to specifically explore the pros and cons of various components of the prototype (eg, color range, explanation of disability, and degree of precision regarding predictive models).

These were shown both to individual patients after their clinical appointment (n=4) as well as to an MS support group (n=11) and patients participating in an MS fundraising walk (n=6). Demographic information was not always collected, but the cohort included both ambulatory and nonambulatory patients, patients seen both within our academic institution and from the broader Northern California community of people living with MS, and also explicitly included patients who self-identified as racial and ethnic minorities. We used the same initial questions as in Textboxes 1 and 2 as well as additional questions from Table 1. After each set of interviews, we incorporated patient feedback into the mock-ups.

**Clinician interview insights to include in the app.**

Include in the app

- How patients compare with those of similar demographics
- Potential treatments
- Data as a function of time
- Better metrics of progression
- Simple visualizations
- Tool to promote communication
- Clarity about the design process

**Clinician interview insights to exclude from the app.**

Exclude from the app

- Information above a high-school level
- Too many numbers or laboratory test data
- Physician feelings and analyses
- Default contextualization (patients should have an option to view how they are doing compared with others)
- Unfiltered scores that patients can misunderstand
- Overly specific information, such as specific risk scores, given the imprecision in data used to generate algorithms

### Phase III—Implementation: Test and Iterate

Our goal in phase III was to develop a minimally viable product (MVP). Therefore, once our phase II mock-ups were refined based on user input; we went through an agile development process to build Open MS BioScreen. We met weekly to review progress of the app and determine how to keep in line with feedback from patients and providers. Phase III meetings were conducted from March 2017 to July 2017. Meetings lasted for 1 hour and focused on app demos and feedback. Open MS BioScreen was built in Ruby [25], a programming language, using a Ruby on Rails development framework, jQuery, and d3.js for the front end. In this phase, the tool itself was submitted for approval by the UCSF institutional review board (IRB), an Information Technology security risk assessment, and privacy legal and risk review. Once we had a final product, we again brought the app to an MS support group, to 15 individual patients, and to advocacy group consultants, to gather and incorporate feedback into the app.

To evaluate the accuracy of basic data entered by participants, in pilot testing, we asked 24 patients who were long-time participants in the UCSF EPIC study [18,19] to enroll in Open MS BioScreen and create a basic profile. After signing a written informed consent for an IRB-approved research protocol, participants enrolled and entered their MS duration, data, and treatments. We then compared these entries to study clinician-entered data available in the EPIC study.

### Ethics Approvals

Funding for this project was provided by the Conrad N. Hilton Foundation. All procedures performed in studies involving human participants were in accordance with the ethical standards of the UCSF IRB and with the 1964 Helsinki declaration and its later amendments or comparable ethical standards. Electronic, implied informed consent was obtained from all individual participants included in the study under UCSF IRB no. 17-22732.

## Results

### Phase I—Inspiration: Empathize and Define

#### Clinician Interviews

Clinicians provided a range of responses regarding key features that would be provided in a tool that engaged patients with MS, allowed them to track their condition in a manner useful to them, share what they track with clinicians, and use visualizations to become more informed participants in their care process (Textboxes 1 and 2). To summarize clinicians’ insights, patient data had to be presented in an understandable format, without excessive details or over-reliance on specific risk ratios or numbers, given the limitations in the data used to generate risk, and the need for visualizations to be inherently meaningful to patients *without* clinician interpretation or input.

#### Patient Interviews

During this phase, 12 patients were interviewed. To summarize their responses, they articulated a need for clarity, simple tracking and display of key MS data, ability to personalize how they viewed their MS course and what factors went into the contextualization algorithm, and positive emphasis on their trajectory and algorithms. For example, if the contextualization algorithm showed that their MS was worsening at a faster rate than others, then a pop-up might encourage them to return to their physical therapist or discuss their optimal MS management with their clinician. These are detailed in Table 1.

#### Comparative Analysis of Existing Patient-Facing Tools

The comparative analysis approach allowed us to identify a number of tools with rich information (illustrative examples given in Table 2) and to use their relative strengths and limitations to inform our own subsequent ideation process. Some of these tools evolved since our original phase I in 2016 and have since been summarized elsewhere [2,26].

On the basis of this analysis, we were able to define several key features for our own ideal platform, namely: (1) web-based and platform-agnostic, that is, not dependent on the life cycle of any other individual technology piece; (2) limited imprint from the pharmaceutical industry; (3) reliable over time without requiring daily input; and (4) clinician-derived informational modules.

### Phase II—Ideation: Ideate and Prototype

#### Mock-Ups

**Figure 3:**
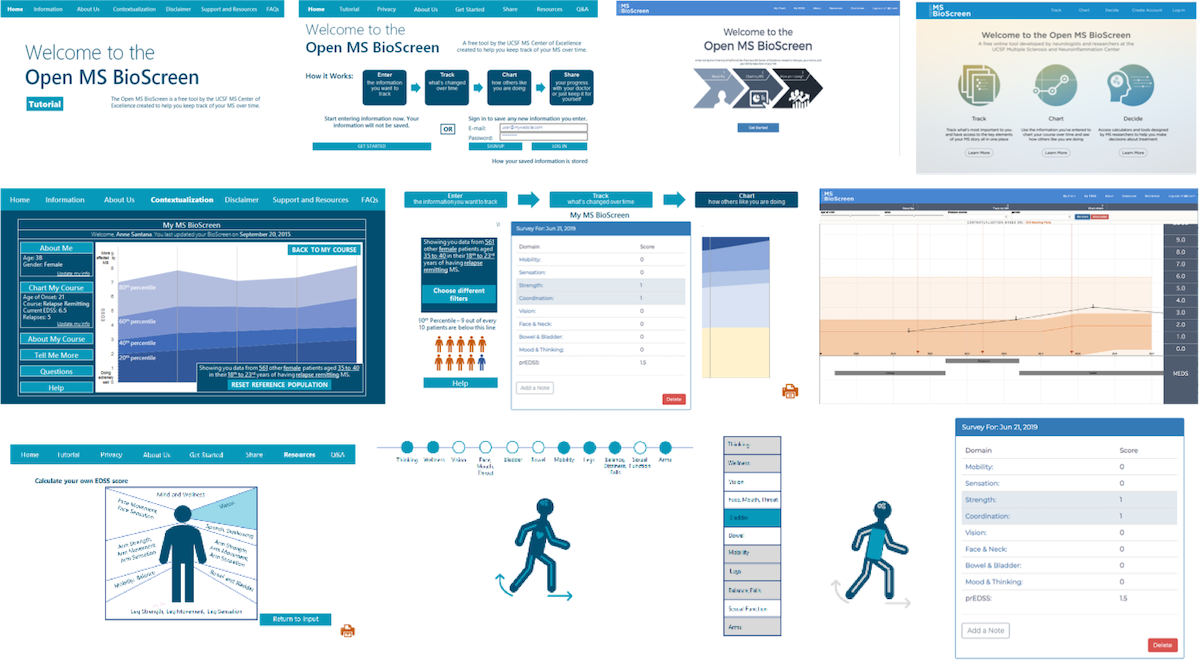
Phase II: Ideation. Evolution of Open MS BioScreen mock-ups. Top panel: landing page. Middle panel: longitudinal disease course visualization and contextualization tool. Bottom panel: assessment and representation of global neurological function.

The priorities identified in the empathize and define phase were used to develop an initial series of mock-ups and click-through presentations of the app (Figure 3). The longitudinal view of the patient course (middle panel, left image) was designed based on the longitudinal view of the original tablet-based MS BioScreen, whereas other features were new. This prototype was then presented to users for feedback on the design and usability of the Open MS BioScreen.

The upper panel demonstrates various iterations of the landing page, which were designed to allow the user to navigate the site with clarity. The middle panel demonstrates a series of iterations of the longitudinal disease contextualization, which was designed to allow the patient to visualize and interpret their clinical course while minimizing the potential for distress. The bottom panel demonstrates iterations of the patient’s global neurological functioning, which was designed to allow patients to enter information about the various functional domains affected by MS (eg, vision, cognition, and walking) and their severity (mild, moderate, or severe), and to visualize these globally. We initially anticipated that patients would want to see their functional domains represented as a sort of avatar, but patients reported that they preferred to have their functional representation alongside, rather than showing their symptoms on a figure. The questions comprising the tool were derived from the prEDSS initially developed by Goodin et al [33], expanded to enable greater sensitivity to changes in cognition and upper limb function over time, and iteratively tested and refined through a series of validation studies (reported separately, manuscript under review). Here, as part of our design work, we specifically iterated visualization of the final prEDSS score but not the questions or scoring.

#### Patient Interviews

With the prototypes, we interviewed another 21 patients. Targeted interviews lasted approximately 15 min each and asked patients to reflect on the clarity of the information, impact (educational or emotional) of the information and visualizations provided, ease of navigation and of data entry when appropriate, and missing aspects of the tool. We recorded all feedback, and then, we developed codes for the responses using a grounded theory approach [34]. The authors (RB and JP) read through all transcripts and identified open codes based on line-by-line analysis of emerging themes. The authors then discussed relationships among open codes and created selective codes, or themes, of larger concepts.

Patient insights were primarily clustered around 6 themes:

1. Information: not knowing which information is important
2. Education: feeling like their doctors are not telling them the whole story
3. Data: wanting more tracking options and the ability to see their own data
4. Emotion: expressions of fear and discretion
5. Clarity: wanting clearer labels and explanations of the tool
6. Perception: wanting the tool to present an action-oriented rather than a pessimistic view of MS progression

### Phase III—Implementation: Test and Iterate

**Figure 4:**
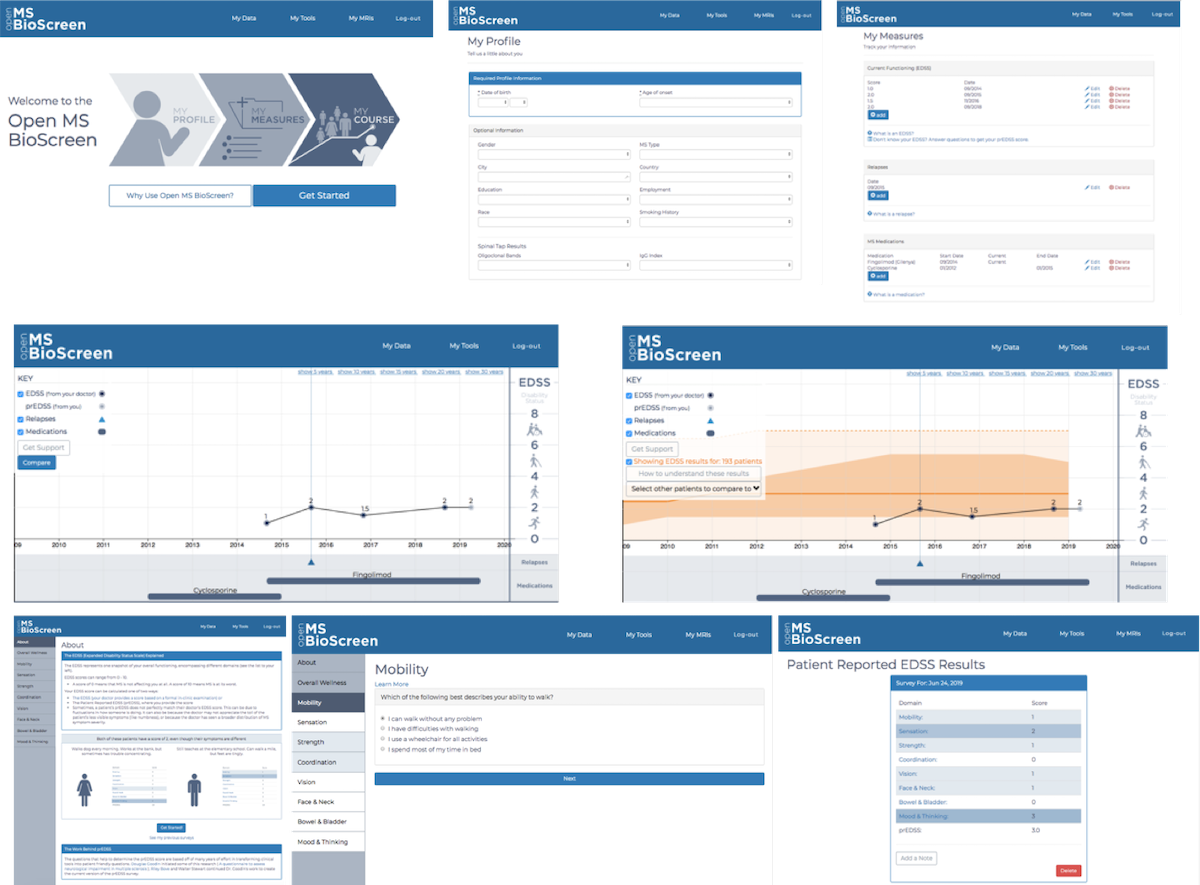
Phase III: Implementation screenshots of Open MS BioScreen’s initial minimally viable product. Top panel: landing page and initial data entry tools. Middle panel: the longitudinal multiple sclerosis course visualization tool, without a contextualization tool. Bottom panel: the patient-reported expanded disability status scale tool, whose development occurred as part of Open MS BioScreen and whose formal clinical validation is reported separately (manuscript under review).

Once phase II was complete, app development of the MVP occurred between March 2017 and September 2017, incorporating insights from phases I and II (Table 3) and turning these into a web-based prototype (Figure 4). We then conducted an additional round of interviews with our 4 stakeholder groups: patients, clinicians, advocacy, and industry representatives. We compiled all the interviews into actionable insights that we incorporated into the MVP (Table 4). This MVP was launched in February 2018 [9].

**Table 3:** Insights derived from 21 patients who viewed mock-ups.

Insight	Examples	Quotes
Information: Patients do not know which information is important (18/21, 86%)	Sharing information across specialties Understanding their course	“If I talk to any other doctor, they don’t know about my specific clinical trial.” “We don’t know which level we're at—so it's important that now that we know how to treat it, now that we have the chance to take better drugs, it's very lucky.”
Education: Patients feel like their doctors are not telling them the whole story (12/21, 57%)	Knowing when their diagnosis changed Understanding the landscape	“I didn't know I was secondary progressive until I decided to read all of the informational packets they give you at the appointment. I said ‘Wait, when did that happen? Nobody told me!’“ “The judgements about who is getting better and who is getting worse, and which drugs are working—because there's no way to truly compare.”
Data: Patients want more tracking options and ability to see their own data (19/21, 91%)	Tracking things that are relevant Comparing their data with other relevant data	“I want a tool that will benefit real people” “There's so many aspects—travel, activity, medicines. I want to see that.” “I'm not curious about other people—usually the information is not relevant because it's so so different for everyone. But this [support group] is a great group, I just wish we were larger.”
Emotion: Patients express fear and discretion (9/21, 43%)	Wanting to not progress or hear about progression	“I don’t want to know if it’s getting worse.” “Sometimes I don't want to know—because I grew up with my father, watching his stages.” “Loved to read, prayed that her vision wouldn't go even if she would be in a wheelchair ”and the lord granted my wish, I'm in a wheelchair, I can read, but I don't read, really anymore. I lost the concentration of what I was reading—I can't remember it.”
Clarity: Patients want clearer labels and explanations of the tool (6/21, 29%)	Understanding the parts of the graph Clarifying the content and labels of the graph	“How is this graph made?” “What is this plotting?” “What do these terms mean?” “It's unclear because there's a chance I'm in one part of the graph, and a chance I'm in another.” “Lack of experience, makes it hard to know where to go.”
Perception: Patients want the tool to present a positive view of multiple sclerosis progression (13/21, 62%)	Tracking full course Understanding treatments and options	“I am living with it, I am not suffering.” “Then they say ‘so and so died from MS,’ and it's like 'Yeah, it's not the MS that kills us, it's the different things that the MS is affecting, but that's only if we sit on our butt and don't do anything.'” “’You'll never be normal’ But I persevered, and it was important to be able to track all my shots, my own things to look out for—I'll know what the things are that I need to do, so I don't fall off the wagon.” “We don't know which level we're at—so it's important that now that we know how to treat it, now that we have the chance to take better drugs, it's very lucky.” “I haven't been seeing anything progressing—but getting better.”

**Table 4:** Examples of actions taken during phase III (implementation) based on patient insights generated in phase 2.

Insights	Actions	Page
Information, Emotion, Clarity	Better explanation of our tool, its origins, and its cost	Tagline on first page
Information, Education, Clarity	Better explanation of how the tool works Bring down perceived barrier for cognitively impaired patients	Chevron design on the front page
Information, Data, Clarity	Easy entry into the tool Clearer navigation	*Get Started* button
Information, Education, Clarity, Perception	Explanation of how to use the tool Provide guidance to the capabilities of the tool	Tutorial page
Information, Education, Emotion, Perception	Clear disclaimer	Edited headers on the Disclaimer page
Information, Education, Clarity	Frequently asked questions and glossary Definitions and explanations page	Questions and Answers page
Information, Education, Data, Clarity, Perception	Information about how this tool uses patient data, and where it came from Better explanation of our mission Better awareness campaign	About Us
Information, Education, Data	Explanation of what is being tracked	Contextualization introduction page
Information, Education, Data, Clarity	Simple data entry Preset options so patients will not have to think of what fields would be useful	Enter your information page
Information, Education, Data, Emotion, Clarity, Perception	More intuitive charts Icons that reflect the words they represent Clearer marking for parts that are important to bring to clinic Explain what it means to “progress”	Clearer design, iterated by patient interactions
Information, Education, Data, Clarity, Perception	Clarity about the difference between your data and the contextualized data of others Clarity about how the curves are generated Careful wording about “similar patients” Filters for demographics	Labels at the top of the screen More explanation about the cohort being compared
Education, Clarity, Emotion	Education about complex topics—EDSS^a^, HIPAA^b^ algorithms, and cohorts	Educational modules
Information, Education, Data, Emotion, Clarity, Perception	Clear labels and instructions for the patient-reported EDSS section	Clearer design, iterated by patient interactions
Information, Education, Data, Emotion, Clarity, Perception	Clearer language Blue-on-white visualization of symptom severity Print-friendly interface	All pages

^a^EDSS: expanded disability status scale.

^b^HIPAA: Health Insurance Portability and Accountability Act.

### Comparison of Basic Clinical Data Collected Through Open MS BioScreen Against Study Clinician–Entered Data

In our pilot testing of 24 patients who were EPIC study participants (median EDSS 2, IQR1-4), we compared patient- and study clinician–entered responses for 3 basic clinical data: MS duration, MS type, and MS disease-modifying therapy (DMT). We found that the mean difference between the patient-reported and study-recorded year of MS onset was −0.29 years (SD 3.16). The MS type included 16 relapsing-remitting (RR), 4 secondary progressive (SP), 2 primary progressive, 2 clinically isolated syndrome (CIS); 83% (20/24) agreed on MS type, with EPIC patient-reported mismatches as follows: SP-RR, SP-RR, RR-SP, and CIS-RR. There was also 83% (20/24) concordance on the most recent DMT; in all residual cases, the patient indicated a newer approved DMT relative to the self-injectable recorded in the database.

### Focus on Accessibility

We prioritized accessibility in a number of ways. We included *open* in the title as a key concept because the platform is freely available for anyone in any location to create a profile and enter their MS measurements, without requiring a fee or any user validation process. Throughout the design process, we worked with end users to make Open MS BioScreen easy to use for different technical skill levels. Open MS BioScreen is currently an English language app, but users could use a web-based translation to view the site in another language, and patients with cognitive or physical limitations can request assistance from a proxy user.

### Added Functionalities

**Figure 5:**
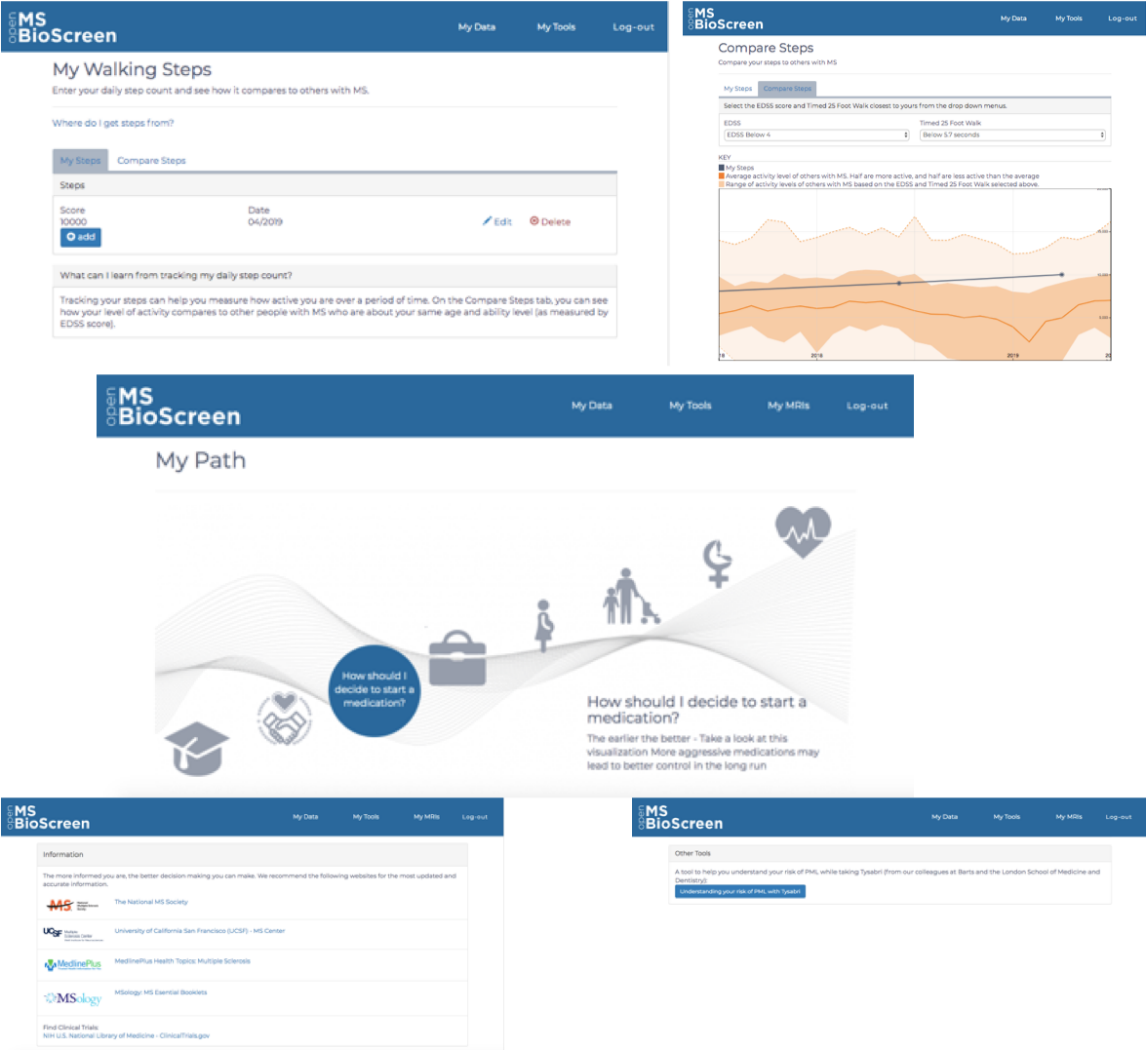
Additional features developed after the Open MS BioScreen live data. The top panel depicts the step count comparison tool that allows patients to enter their daily step count and compare this with others from a cohort of patients with multiple sclerosis (MS) at their phase of the disease [35]. The middle panel depicts My MS Paths, a visualization narrating specific decisions and challenges that patients may face over their lifetime living with MS. The bottom panel shows links to algorithms and clinical decision support tools developed by other investigators that provided complementary information and for which links were provided on Open MS BioScreen.

In addition to incorporating user feedback into the MVP, we also built Open MS BioScreen in such a way that we could easily edit existing functionality or add new features. We focused on three initial types of tools, as illustrated in Figure 5.

As an example of a tool that allows patients to record and track their wearable device data in an effort to address the known limitations of existing tools such as the EDSS [36], we built a tool that allows users to manually enter their step count (as reported by any commercially available wearable device) and to compare their count with disability level- and walking speed–matched patients from a research cohort of 100 UCSF patients with MS followed for over 1 year [13,14,35]. Aside from the data type check, there is no data validation done on the Open MS BioScreen side.

As an example of a tool designed to provide personalized guidance to patients with MS at various points over their life course, we built My MS Paths, a visualization narrating specific decisions and challenges that patients may encounter.

As an example of a clinical decision support tool developed and validated by other investigators but felt to provide significant value to patients with MS, we created a series of links on Open MS BioScreen to sites that allowed patients to calculate their risk, for example, of developing a fatal brain infection, progressive multifocal leukoencephalopathy, while on natalizumab treatment [37].

Again, we worked with end users and our development team to design and build these features. Due to our flexible platform and agile development process, we were able to go live with features as soon as the research was published; this allows patients to have access to actionable research immediately. At the time of manuscript submission, additional features are in development.

## Discussion

### Strengths and Limitations

We detail a human-centered approach undertaken to engage stakeholders in the iterative development of an on-demand tool designed to expand patient access to clinically actionable research insights generated by MS researchers and academics. A major limitation of the original MS BioScreen tablet-based app was that it did not take into account many of these principles, and as a result, it was not accessible, agile, or designed with user (patient and clinician) experience in mind. Here, we prioritized the patient and clinician’s experience, carefully assessing for similar tools that might be available commercially or not-for-profit, and returned to stakeholders at each step of the development process to ensure that the end result was understandable and actionable. We were able to achieve the 4 main features articulated during our ideation process, features that to our knowledge were not all present in any existing tool reviewed: (1) web-based and platform-agnostic, that is, not dependent on the life cycle of any other individual technology piece; (2) limited imprint from the pharmaceutical industry; (3) reliable over time without requiring daily input; and (4) clinician-derived informational modules.

The current platform provides a number of benefits to patient users. It allows patients to enter and track regular changes in their MS course, including important treatment changes, relapses, and changes in clinical function. As these data are available live in Open MS BioScreen, as opposed to a health system’s electronic medical record, patients can easily maintain continuity over time, across providers and health systems. This is beneficial in health care settings such as the United States, where, in contrast to countries such as Sweden that benefit from a central, integrated MS registry [29], patients must navigate a number of platforms and systems—with the potential for data loss, data duplication, and burdensome navigation of EHRs to synthesize the relevant MS-related information as patients transition between specialists and care settings. Of note, within UCSF, we have built a platform, BRIDGE, that launches directly from the EHR (bridge.ucsf.edu). Open MS BioScreen also allows patients to access narrative visualizations through which they can explore current and next phases in their clinical trajectory with MS, such as how to select an initial DMT, how to interpret and respond to a diagnosis of clinical progression, and how to approach upcoming life transitions (eg, pregnancy, menopause, or healthy aging). It also allows them to develop personalized action plans based on other patients living with MS (rather than the general population), taking into account realistic goal setting for their daily activity levels. The next phase of development is to measure and monitor the uptake of the tool in response to planned dissemination efforts and to continue to fine tune features based on user input.

The current platform also prioritizes certain clinician and researcher needs. First, Clinicians meeting a patient with long-standing MS must often spend valuable encounter time reviewing copious historical and administrative records to extract key MS-related clinical information. Second, Neurologists who are not MS experts also benefit from benchmarking a given patient’s current function against the UCSF MS research cohort, a form of *virtual cohort* to inform expectations about patient course. Third, patients who are more informed about treatment decisions, such as weighing risks and benefits of therapies or what to expect at various life stages, are better able to participate in the clinical decision-making process. Over time, the research community will benefit from these data (which patients explicitly consent to be shared in a deidentified manner), to monitor trends in patient-related function, information-seeking, adaptation, and interpretation of data from wearable technology, and eventually MRI analytics. One obvious limitation, for both clinicians and researchers, is that there is no formal validation process for user-entered data, although our pilot testing for 24 participants suggests reasonable agreement between patients and clinicians regarding key MS metrics.

### Conclusions

A major recommendation from patients, industry, and advocacy stakeholders was the need to incorporate a greater number of patient-generated data, such as ambulatory activity and MRIs. Unfortunately, the field of commercially available wearable devices is rapidly evolving in terms of both device engineering as well as regulations surrounding devices and data use. Given this landscape, rather than invest substantially in developing Application Programming Interfaces with selected vendors and industry partners (who may or may not retain market share) to synchronize data from wearables, we focused instead on a simpler, more agile MVP. We, therefore, designed a simple, device-agnostic solution that enables patients with MS to enter data about their daily step count obtained from any number of commercial activity trackers and to obtain contextualizations of their ambulatory activity according to their own age and ability level. Our initial platform will allow for agile and responsive development and flexible adaptation of clinically actionable algorithms and tools developed by other research groups. This will be instrumental in ensuring that Open MS BioScreen allows patients to monitor their own course and make informed decisions based on current advances in clinical research.

As with any digital tool, there are a number of sustainability concerns that will be navigated, for which we have created a dissemination and sustainability roadmap. Dissemination and adaptation of digital tools represent a significant next phase in Open MS BioScreen development. Planned analysis of this dissemination plan includes a 2-year analysis of Google Analytics user activity data to track the dissemination and adaptation of this platform by patients living outside the UCSF research space. We will also include direct user feedback through the platform itself as well as through an additional cycle of interviews with users after the completion of the next modules (MRI data). These planned sources of feedback will allow us to expand within the inevitable biases of any specific academic, clinical, or geographic setting. Although we took care throughout our design process to interview a range of patients and clinicians both within and outside the UCSF MS group, there are likely residual biases that will be addressed through this planned feedback process. Financially, beyond the current philanthropy (Conrad N. Hilton Foundation) funding cycle, the dissemination results will allow us to determine if the platform is best maintained within an academic setting or would benefit from the adaptation by a nonprofit partner.

To date, there have been limited descriptions of human-centered design in MS care [2,38-42]. The insights gained from our extensive human-centered design process described here can inform approaches for developing a number of tools that enable tracking, communicating, and eventually improving function in patients with chronic conditions characterized by variable course, functional impairments, and a strong focus on quality of life.

## Appendix

Multimedia Appendix 1 Patient interview questions used in phase I.

Multimedia Appendix 2 Patient interview questions used in phase II.

## Abbreviations

CIS: clinically isolated syndrome
DMT: disease-modifying therapy
EDSS: expanded disability status scale
EHR: electronic health record
EPIC: expression, proteomics, imaging, clinical longitudinal study
IRB: institutional review board
MRI: magnetic resonance imaging
MS: multiple sclerosis
MVP: minimally viable product
NMSS: National Multiple Sclerosis Society
prEDSS: patient-reported expanded disability status scale
RR: relapsing-remitting (multiple sclerosis)
SP: secondary progressive (multiple sclerosis)
UCSF: University of California, San Francisco

## References

[1] Hauser SL, Goodin D, Jameson JL, Fauci A, Hauser S, Longo D, Kasper D, Loscalzo J (2012). Multiple sclerosis and other demyelinating diseases. Harrison's Principles of Internal Medicine.

[2] Giunti G, Fernández EG, Zubiete ED, Romero OR (2018). Supply and demand in mhealth apps for persons with multiple sclerosis: systematic search in app stores and scoping literature review. JMIR Mhealth Uhealth.

[3] McBurney R, Zhao Y, Loud S, Balasubramanian R, Schmidt H, Kolaczkowski L (2017). Initial Characterization of Participants in the iConquerMS Network. Proceedings of the Americas Committee for Treatment and Research in Multiple Sclerosis.

[4] Vollmer TL, Ni W, Stanton S, Hadjimichael O (1999). The NARCOMS patient registry: a resource for investigators. Int J MS Care.

[5] Wicks P, Massagli M, Frost J, Brownstein C, Okun S, Vaughan T, Bradley R, Heywood J (2010). Sharing health data for better outcomes on PatientsLikeMe. J Med Internet Res.

[6] Hill E (2014). Smart patients. Lancet Oncol.

[7] Househ M, Borycki E, Kushniruk A (2014). Empowering patients through social media: the benefits and challenges. Health Informatics J.

[8] Gourraud P, Henry RG, Cree BA, Crane JC, Lizee A, Olson MP, Santaniello AV, Datta E, Zhu AH, Bevan CJ, Gelfand JM, Graves JS, Goodin DS, Green AJ, von Büdingen HC, Waubant E, Zamvil SS, Crabtree-Hartman E, Nelson S, Baranzini SE, Hauser SL (2014). Precision medicine in chronic disease management: the multiple sclerosis BioScreen. Ann Neurol.

[9] Open MS BioScreen.

[10] (2015). IDEO.

[11] Matheson GO, Pacione C, Shultz RK, Klügl M (2015). Leveraging human-centered design in chronic disease prevention. Am J Prev Med.

[12] Yu CH, Parsons JA, Mamdani M, Lebovic G, Hall S, Newton D, Shah BR, Bhattacharyya O, Laupacis A, Straus SE (2014). A web-based intervention to support self-management of patients with type 2 diabetes mellitus: effect on self-efficacy, self-care and diabetes distress. BMC Med Inform Decis Mak.

[13] Karmali KN, Davies P, Taylor F, Beswick A, Martin N, Ebrahim S (2014). Promoting patient uptake and adherence in cardiac rehabilitation. Cochrane Database Syst Rev.

[14] Altman M, Huang TT, Breland JY (2018). Design thinking in health care. Prev Chronic Dis.

[15] Cristancho-Lacroix V, Moulin F, Wrobel J, Batrancourt B, Plichart M, de Rotrou J, Cantegreil-Kallen I, Rigaud A (2014). A web-based program for informal caregivers of persons with Alzheimer's disease: an iterative user-centered design. JMIR Res Protoc.

[16] Velardo C, Shah SA, Gibson O, Clifford G, Heneghan C, Rutter H, Farmer A, Tarassenko L, EDGE COPD Team (2017). Digital health system for personalised COPD long-term management. BMC Med Inform Decis Mak.

[17] Trail-Mahan T, Heisler S, Katica M (2016). Quality improvement project to improve patient satisfaction with pain management: using human-centered design. J Nurs Care Qual.

[18] Cree BA, Gourraud P, Oksenberg JR, Bevan C, Crabtree-Hartman E, Gelfand JM, Goodin DS, Graves J, Green AJ, Mowry E, Okuda DT, Pelletier D, von Büdingen HC, Zamvil SS, Agrawal A, Caillier S, Ciocca C, Gomez R, Kanner R, Lincoln R, Lizee A, Qualley P, Santaniello A, Suleiman L, Bucci M, Panara V, Papinutto N, Stern WA, Zhu AH, Cutter GR, Baranzini S, Henry RG, Hauser SL, University of California‚ San Francisco MS-EPIC Team (2016). Long-term evolution of multiple sclerosis disability in the treatment era. Ann Neurol.

[19] University of California San Francisco.

[20] Pletcher MR, Robinson A, Najafi N, Patel S, Franzon D, Bove R (2019). How Do We Deliver AI in Real Time to Clinicians?. Proceedings of the UC-Wide AI in Biomedicine Conference.

[21] Shah SG, Robinson I (2008). Medical device technologies: who is the user?. Int J Healthc Tech Manag.

[22] Kurtzke JF (1983). Rating neurologic impairment in multiple sclerosis: an expanded disability status scale (EDSS). Neurology.

[23] (2019). Balsamiq. Rapid, Effective and Fun Wireframing Software.

[24] (2019). InVision | Digital Product Design, Workflow & Collaboration.

[25] (2019). Ruby.

[26] Stoll S, Litchman T, Wesley S, Litchman C (2019). Multiple sclerosis apps: the dawn of a new era: a comprehensive review. Neurology.

[27] Irody I (2019). AppAdvice.

[28] Pelletier D, Rennert T, Chang M, Decunto S, Cen S (2019). myMS; a comprehensive patient-centered mobile app to monitor MS at home (P3.2-003). Neurology.

[29] Hillert J, Stawiarz L (2015). The Swedish MS registry–clinical support tool and scientific resource. Acta Neurol Scand.

[30] Genentech I (2019). Floodlight Open. Understanding MS Together.

[31] (2019). The Aby App.

[32] (2019). BeCareLink: Connecting Data, Medicine & Technology.

[33] Goodin DS (1998). A questionnaire to assess neurological impairment in multiple sclerosis. Mult Scler.

[34] Glaser BG, Strauss AL (2017). The Discovery of Grounded Theory.

[35] Block VJ, Bove R, Zhao C, Garcha P, Graves J, Romeo AR, Green AJ, Allen DD, Hollenbach JA, Olgin JE, Marcus GM, Pletcher MJ, Cree BAC, Gelfand JM (2019). Association of continuous assessment of step count by remote monitoring with disability progression among adults with multiple sclerosis. JAMA Netw Open.

[36] Hobart JC, Cano SJ, Zajicek JP, Thompson AJ (2007). Rating scales as outcome measures for clinical trials in neurology: problems, solutions, and recommendations. Lancet Neurol.

[37] Major EO, Yousry TA, Clifford DB (2018). Pathogenesis of progressive multifocal leukoencephalopathy and risks associated with treatments for multiple sclerosis: a decade of lessons learned. Lancet Neurol.

[38] Berry AB, Butler KA, Harrington C, Braxton MO, Walker AJ, Pete N, Johnson T, Oberle MW, Haselkorn J, Paul Nichol W, Haselkorn M (2016). Using conceptual work products of health care to design health IT. J Biomed Inform.

[39] Giunti G, Kool J, Romero OR, Zubiete ED (2018). Exploring the specific needs of persons with multiple sclerosis for mhealth solutions for physical activity: mixed-methods study. JMIR Mhealth Uhealth.

[40] Giunti G, Mylonopoulou V, Romero OD (2018). More stamina, a gamified mhealth solution for persons with multiple sclerosis: research through design. JMIR Mhealth Uhealth.

[41] Thirumalai M, Rimmer JH, Johnson G, Wilroy J, Young H, Mehta T, Lai B (2018). TEAMS (tele-exercise and multiple sclerosis), a tailored telerehabilitation mhealth app: participant-centered development and usability study. JMIR Mhealth Uhealth.

[42] Thomas S, Pulman A, Thomas P, Collard S, Jiang N, Dogan H, Smith AD, Hourihan S, Roberts F, Kersten P, Pretty K, Miller JK, Stanley K, Gay M (2019). Digitizing a face-to-face group fatigue management program: exploring the views of people with multiple sclerosis and health care professionals via consultation groups and interviews. JMIR Form Res.

