# Supplementary material for: A Precision Medicine Tool for Patients With Multiple Sclerosis (the Open MS BioScreen): Human-Centered Design and Development
Source: J Med Internet Res. 2020 Jul 6;22(7):e15605. doi: 10.2196/15605 (PMC7381029; doi:10.2196/15605)
Supplement: Multimedia Appendix 1 [file jmir_v22i7e15605_app1.docx]

**Multimedia Appendix 1**: Patient interview questions used in Phase 1

| Lead Statement/Question | Follow-up Question |
| --- | --- |
|  |  |
| **May I ask a few questions about your experience with MS?** |  |
| How old were you when you were diagnosed with MS? | How old are you now? |
| What kind of MS do you have? |  |
| **These next few questions ask about information that you may get from your doctor, keep track of or find on your own.** |  |
| At the end of your doctor appointments, does the doctor or medical assistant give you a summary - either written or electronic? | If yes… |
|  | How useful is it? |
|  | What do you find useful? Why? |
|  | What is not useful about it? Why? |
| In between visits to your doctor, do you keep track of any information about your MS? | If yes… |
|  | What information do you track? |
|  | Why do you track this? |
|  | How do you track it? (Paper, computer, smart phone?) |
| In between visits to your doctor, do you ever look up information about your MS? | If yes… |
|  | Where do you look for information? |
|  | What kinds of information do you look up? |
|  | What do you do with the information you look up? |
|  | Do you share this information with anyone? |
|  | How do you share it? |
