# Supplementary material for: A Precision Medicine Tool for Patients With Multiple Sclerosis (the Open MS BioScreen): Human-Centered Design and Development
Source: J Med Internet Res. 2020 Jul 6;22(7):e15605. doi: 10.2196/15605 (PMC7381029; doi:10.2196/15605)
Supplement: Multimedia Appendix 2 [file jmir_v22i7e15605_app2.docx]

**Multimedia Appendix 2**: Patient interview questions used in Phase 2

| Lead Statement/Question | Follow-up Question |
| --- | --- |
|  |  |
| **These last few questions ask about information you may find helpful or interesting.** |  |
| Researchers at UCSF are creating a secure website that can help you track how you are doing over time. | Would you be interested in tracking how you are doing and seeing it trended over time? |
|  | What are some things you would want to track? |
| This website will also be able to show you how other patients like you are doing -- patients your age who've had MS the same length of time. | Would you be interested in a seeing how other MS patients like you are doing in terms of mobility? |
|  | Why? |
| **My final two questions are very general.** |  |
| How confident are you filling out medical forms by yourself? |  |
| How often do you use a computer? |  |
